# Advancing prognostic assessment in primary sclerosing cholangitis: insights from abdominal magnetic resonance imaging and magnetic resonance cholangiopancreatography with Anali scores

**DOI:** 10.1590/0100-3984.2023.56.6e2

**Published:** 2023

**Authors:** Renato Jose Kist de Mello, Alice Schuch

**Affiliations:** 1 Radiologist, member of the Abdominal Radiology Group at the Hospital Moinhos de Vento and at the Hospital Santa Casa de Porto Alegre, Porto Alegre, RS, Brazil. Email: renato.jkm@gmail.com.; 2 Abdominal Radiologist, Head of the Department of Radiology and Diagnostic Imaging at the Hospital Moinhos de Vento, Porto Alegre, RS, Brazil. Email: alice.schuch@hmv.org.br.

Primary sclerosing cholangitis (PSC) is a rare chronic cholestatic liver disease with an overall annual incidence rate of 0.77 per 100,000 population^(1)^. It is characterized by chronic inflammation of the bile ducts, which may lead to complications such as cirrhosis and neoplasia (cholangiocarcinoma). Contrast-enhanced magnetic resonance imaging (MRI) of the abdomen and magnetic resonance cholangiopancreatography, together with clinical and laboratory findings, are essential for the noninvasive diagnosis and surveillance of PSC. Invasive procedures, like endoscopic retrograde cholangiopancreatography, are less accessible and have potential severe complications, such as duodenal perforation and pancreatitis.

The Anali score was developed in 2014 to predict the prognosis of patients with PSC^(1)^. It was based on the combination of predictive MRI features without and with intravenous gadolinium administration. The Anali score without gadolinium ranges from 0 to 5. Of those five points, two refer to the evaluation of sclerosing cholangitis, by grading the dilatation of the biliary tract (based on the measurement of its maximum diameter: as 0 points if it was ≤ 3 mm, 1 point if it was 3–5 mm, and 2 points if it was ≥ 5 mm); and three of the points refer to signs of chronic liver disease (2 for hepatic dysmorphism and 1 for portal hypertension). The Anali score with gadolinium ranges from 0 to 2 (based on dysmorphism and the heterogeneity of parenchymal enhancement). Both Anali scores have been associated with radiological progression of PSC. The evaluation of hepatic morphology and signs of heterogeneous peribiliary enhancement^(2)^, as well as that of portal hypertension, plays an important role in assessing the risk of PSC and does not depend exclusively on the degree of bile duct dilatation.

The study conducted by López Grove et al.^(3)^ and published in this issue of **Radiologia Brasileira** focuses on the importance of contrast-enhanced MRI of the abdomen and magnetic resonance cholangiopancreatography for the noninvasive investigation of patients with PSC and for predicting disease progression, using the Anali scores together with clinical outcomes, such as liver transplantation, decompensation of liver cirrhosis, and death. Two other MRI signs were used for the PCS evaluation: periportal edema and heterogeneous liver parenchymal signal on diffusion-weighted imaging sequences, which are routinely obtained during contrast-enhanced MRI examinations of the upper abdomen. Another significant result was the high level of interobserver agreement. Because of the variability of data and the frequent monitoring of the status of patients with PSC, it is very important to use objective radiology scores in the imaging routine for such patients. An objective assessment of the signs of PSC would surely simplify the work of radiologists and clinicians in the continuous evaluation of the patients.

The López Grove et al.^(3)^ study represents the first analysis of Anali scores in South America. It is crucial to confirm the external validity of the Anali scores, as well as to promote their widespread recognition and acceptance among radiologists. Further studies are needed in order to identify the early signs of hepatic inflammation that could lead to fibrosis in patients with PSC, such as liver stiffness^(4)^. Similarly, the assessment and prognosis of patients diagnosed with nonalcoholic steatohepatitis, which is another type of liver inflammation, could benefit from liver stiffness measurement.
